# Sentinel Surveillance of Influenza A in Libya: Subtyping and Genomic Analysis During Recent Seasons (2022–2024)

**DOI:** 10.3390/tropicalmed11050127

**Published:** 2026-05-08

**Authors:** Mahmud Azbida, Sana Ferjani, Omar Elahmer, Rmadhan Osman, Salem Shenaisheh, Amal Barakat, Salma Abid, Adem Eljerbi, Abdulwahab Kammon, Ameni Sallemi, Haider El-Saeh, Ilhem Boutiba-Ben Boubaker, Ibrahim Eldaghayes

**Affiliations:** 1 Department of Medical Laboratories, Faculty of Medical Technology, University of Tripoli, Tripoli 13275, Libya; 2 Department of Microbiology, Charles Nicolle Hospital, Tunis 1006, Tunisia; 3 LR99ES09AMR Research Laboratory, Faculty of Medicine of Tunis, University of Tunis ElManar, Tunis 1007, Tunisia; 4 National Center for Disease Control, Tripoli 71171, Libya; 5 Department of Dental Technology, Faculty of Medical Technology, University of Tripoli, Tripoli 13275, Libya; 6 Department of Public Health, Faculty of Medical Technology, University of Tripoli, Tripoli 13275, Libya; 7 Community and Family Medicine Department, Faculty of Medicine, University of Misurata, Misurata 2478, Libya; 8 Regional Office for the Eastern Mediterranean, World Health Organization, Cairo 7608, Egypt; 9 National Research Centre for Tropical and Transboundary Diseases, Alzintan 61053, Libya; 10 Laboratory of Clinical Pharmacology, National Center Chalbibelkahia of Pharmacovigilance of Tunis, 9 Avenue Dr. Zouhaier Essafi 1006 Bab Souika, Tunis 1007, Tunisia; 11 Department of Microbiology and Parasitology, Faculty of Veterinary Medicine, University of Tripoli, Tripoli 13662, Libya; 12 Influenza Focal Point, National Center for Disease Control, Tripoli 71171, Libya

**Keywords:** influenza A virus, molecular surveillance, subtyping, genome sequencing, phylogenetic analysis, sentinel surveillance, Libya

## Abstract

Influenza sentinel surveillance in Libya was formally established in 2022 by the Libyan National Center for Disease Control (NCDC). Between 2022 and 2024, a total of 1864 nasopharyngeal specimens were collected from patients presenting with influenza-like illness and tested using the GeneXpert for influenza A virus, influenza B virus, severe acute respiratory syndrome coronavirus 2 (SARS-CoV-2), and respiratory syncytial virus (RSV). Influenza A virus was detected in 21.1% (393/1864) of samples and influenza B virus was detected in 5.4% of samples (100/1864). SARS-CoV-2 and RSV were identified in 11.6% (216/1864) and 4.1% (77/1864) of specimens, respectively. A subset of 22 influenza A-positive samples was selected based on sample availability and sufficient remaining volume after the initial test for confirmatory testing and further molecular characterization. Real-time RT-PCR subtyping identified 11 A(H1N1)pdm09 and four A(H3N2) viruses. Whole-genome sequencing was successfully performed for 11 isolates, followed by phylogenetic analysis. Genetic characterization revealed that all A(H1N1)pdm09 viruses belonged to clade 6B.1A.5a.2a (5a.2a), while A(H3N2) viruses clustered within clade 3C.2a1b.2a.2a.3a.1 (2a.3a.1) were based on hemagglutinin gene mutations. No neuraminidase mutations associated with antiviral resistance were detected. This study represents the first molecular and phylogenetic characterization of circulating human influenza viruses in Libya, with sequence data submitted to the Global Initiative on Sharing All Influenza Data (GISAID) to establish baseline genetic data for influenza viruses in Libya.

## 1. Introduction

Globally, respiratory infections remain a leading cause of mortality, with influenza viruses representing major contributors to seasonal viral respiratory epidemics [1]. Among the four types of influenza viruses—A, B, C, and D—influenza A viruses (IAVs) are the most frequently detected and are responsible for the majority of influenza epidemics in humans [2]. Seasonal influenza represents a substantial global disease burden, with approximately one billion infections occurring each year, of which 3 to 5 million are classified as severe, resulting in an estimated 290,000 to 650,000 deaths annually. Reducing the impact of seasonal influenza through improved surveillance, prevention, and control helps countries prepare for future pandemics [1]. Influenza virus infections cause an acute respiratory illness primarily affecting the airways and are characterized by symptoms including fever, dry cough, headache, chills, and sore throat [3].

IAVs are categorized into multiple subtypes based on the presence of two surface glycoproteins, hemagglutinin (HA) and neuraminidase (NA), which are primarily found in wild birds. To date, IAVs have been classified into 18 HA subtypes (H1–H18) and 11 NA subtypes (N1–N11), which can combine in various ways to produce subtypes such as H1N1, H3N2, and others [2]. IAVs belong to the family Orthomyxoviridae. Their genome consists of eight segments of negative-sense single-stranded RNA, with a total length of approximately 13,000 nucleotides, encoding at least 12 viral proteins [4].

Historically, influenza pandemics have occurred at irregular intervals, with their emergence and impact shaped by complex ecological and epidemiological factors. The most severe of these was the 1918 influenza pandemic (Spanish flu), caused by influenza A viruses (IAVs), which infected approximately one-third of the global population and led to an estimated 50–100 million deaths [5,6]. Subsequent pandemics, including the 2009 influenza A(H1N1)pdm09 outbreak, further demonstrated the continuing global threat posed by influenza viruses. In Libya, the first cases of influenza A(H1N1)pdm09 were detected in July 2009 through the surveillance system of the Libyan National Center for Disease Control (NCDC) [7].

In the Northern Hemisphere, influenza activity typically peaks between January and March [8]. As Libya is located in the Northern Hemisphere, it is expected to follow a similar seasonal influenza pattern [1,9]. Although several mechanisms have been proposed to explain the seasonality of influenza in humans—including climatic factors, human behavioral patterns, and the effects of environmental changes on the immune system—the seasonal dynamics of respiratory viruses remain incompletely understood [10].

IAVs possess a well-established capacity to generate new subtypes through rapid genetic changes, which underlie their potential for global spread. Understanding their genetic evolution is therefore essential for predicting outbreak risks and guiding the development of effective vaccines and antiviral therapies [11]. According to the World Health Organization, A(H1N1)pdm09 was the most active influenza A subtype in North Africa between September 2023 and January 2024 [12].

Despite extensive global surveillance efforts, significant challenges persist because IAVs continuously evolve, often reducing vaccine effectiveness when antigenic mismatches occur [13]. Consequently, a comprehensive understanding of influenza A virus (IAV) genetic diversity is critical for optimizing vaccine design and strengthening future pandemic preparedness [14]. Whole-genome sequencing plays a crucial role in detecting novel IAV reassortants and characterizing their molecular properties, including host range and virulence [15]. Although viral RNA stability can be affected by prolonged storage and repeated freeze–thaw cycles [16], all samples included in this study were handled and stored under appropriate conditions to preserve RNA integrity.

In modern molecular epidemiology, phylogenetic analysis has become an indispensable tool for tracking the evolutionary dynamics and transmission pathways of seasonal influenza viruses. The use of real-time genomic surveillance platforms, such as Nextstrain, facilitates rapid sharing and visualization of analyses for important pathogens, including influenza A(H1N1)pdm09 and A(H3N2) [17].

The aim of this study was to detect, subtype, and sequence IAVs in human samples collected through the NCDC sentinel surveillance system in Libya, during recent influenza seasons, in order to address the existing gap in influenza genomic data from the country.

## 2. Materials and Methods

### 2.1. Influenza Surveillance in Libya

**Figure 1:**
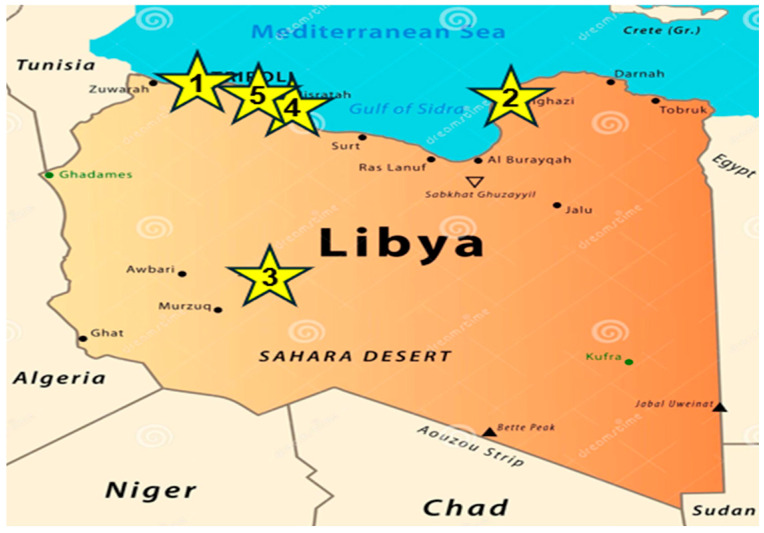
Influenza sentinel surveillance sites in Libyan cities (1: Tripoli [5 sites]; 2: Benghazi [3 sites]; 3: Sebha [3 sites]; 4: Misrata [2 sites]; 5: Zliten [2 sites]).

The NCDC established an influenza sentinel surveillance program in 2022, initially comprising a single sentinel site in Tripoli. Over time, the network had expanded to 15 sentinel sites across five cities in Libya (Figure 1). The full network of 15 sentinel units was not active throughout the study period. During 2022–2024, active surveillance was conducted in Tripoli (5 sites), Benghazi (3 sites), and Sabha (3 sites). Although the network was expanded to include Misrata and Zliten, these additional units were only established and staff trained in 2025. Due to the lack of funding and unavailability of diagnostic kits, no systematic sample collection was conducted in 2025.

### 2.2. Collection of Samples

**Figure 2:**
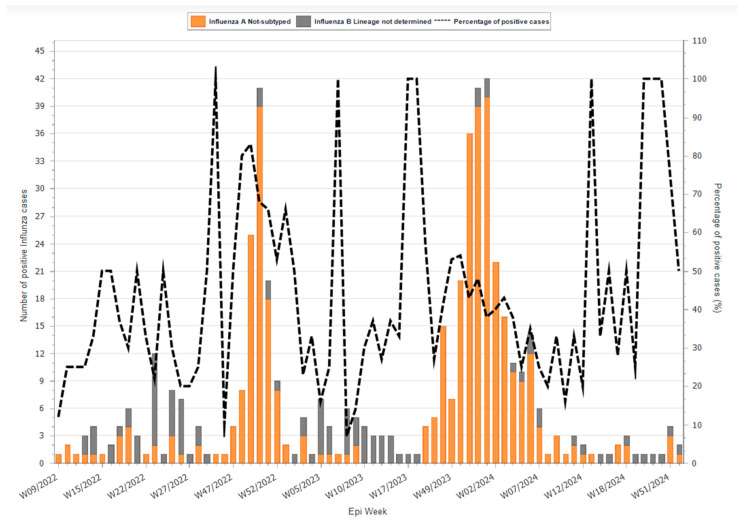
Weekly number of influenza type A- and B-positive cases (bars) and percentage of influenza-positive samples (line) among tested samples in Libya during 2022–2024. (Figure created by Eastern Mediterranean Flu Network (EMFLU, WHO)).

A total of 1864 clinical samples were obtained from patients meeting the WHO case definitions for influenza-like illness (ILI) and/or severe acute respiratory infection (SARI) as part of the routine sentinel surveillance program conducted by the NCDC across influenza sentinel sites in Libya. Samples were collected during the influenza seasons from early 2022 to the end of 2024. At each sentinel site, a limited number of samples were collected during the first three days of each week, following standardized surveillance protocols. Nasopharyngeal swabs were placed in transport media, stored at 2–8 °C, and transported to the laboratory within 72 h for testing. All influenza-positive samples were subsequently stored at −80 °C to preserve RNA integrity. The weekly distribution of influenza A- and B-positive cases and the percentage of positive samples during the study period are presented in Figure 2.

### 2.3. Laboratory Analysis

A total of 1864 nasopharyngeal samples were tested using the GeneXpert system at sentinel site laboratories to detect influenza A, influenza B, SARS-CoV-2, and RSV. The GeneXpert assay (Xpert Xpress Flu/RSV; Cepheid, Sunnyvale, CA, USA) used in this study is a cartridge-based, real-time RT-PCR system in which the exact primer and probe sequences are proprietary and therefore not publicly disclosed by the manufacturer.

From the influenza A-positive samples, 22 samples were selected for further analysis based on sample availability and sufficient remaining volume after initial testing. Only samples that remained suitable for downstream molecular analysis after storage were included. One milliliter from each selected sample was used to confirm the presence of influenza A virus at the National Research Center for Tropical and Transboundary Diseases (NRCTTD). Viral RNA was extracted using the Sterilab NC-15 Plus Automated System with the Alpha & Gene extraction kit (HanwoolTPC Co., Ltd., Bucheon-si, Gyeonggi-do, Republic of Korea), followed by real-time RT-PCR using the Long Gene system, according to the manufacturer’s instructions.

The remaining aliquots were transferred to the National Influenza Center in Tunisia for further confirmation, subtyping, and sequencing. RNA was extracted using the QIAGEN EZ1 Connect MDx system with the EZ1 DSP Virus Kit, and RNA quantity and quality were assessed using a Qubit Fluorometer. Real-time RT-PCR was performed using the Applied Biosystems 7500 Fast system (Applied Biosystems, Thermo Fisher Scientific, Waltham, MA, USA) with the CDC rRT-PCR Influenza Panel. Subtyping was conducted using the CDC Influenza Virus RT-PCR Subtyping Panel.

RNA libraries were prepared using the Illumina RNA Prep with Enrichment kit and sequenced on the Illumina iSeq 100 platform (Illumina Inc., San Diego, CA, USA). Only samples with sufficient RNA quality after storage and transport were included in sequencing. Influenza B-positive samples were not sequenced as the focus was on the type A influenza virus.

### 2.4. Bioinformatic Analysis

Following completion of the Illumina iSeq 100 sequencing run, raw sequencing data were converted into FASTQ files using Illumina Local Run Manager (Illumina Inc., San Diego, CA, USA). Sequencing was performed using paired-end reads (2 × 110 bp). Genome Detective (Pan-viral, v2.21.4) was used to perform read quality control, including trimming of low-quality bases and removal of short reads. Filtered reads were then subjected to de novo assembly and reference-based alignment against IAV reference genomes [18].

Consensus genome sequences were generated based on majority nucleotide calling, with positions of low coverage carefully evaluated. The resulting consensus sequences were exported in FASTA format and submitted to the GISAID EpiFlu™ database. Mutation analysis was performed using FluSurver (https://gisaid.org/database-features/flusurver-mutations-app/ (accessed on 15 March 2026)) (GISAID) to identify nucleotide and amino acid substitutions related to reference influenza A strains.

Phylogenetic analyses were conducted based on the HA gene. Closely related reference sequences were selected using the Audacity Instant tool in GISAID. Two datasets were generated: 118 HA1 sequences (https://doi.org/10.55876/gis8.260101zo) and 58 HA3 sequences (https://doi.org/10.55876/gis8.260101fs). All data were used in accordance with GISAID terms. Phylogenetic and temporal analyses were performed using the Nextstrain pipeline [19].

## 3. Results

### 3.1. Detection of Influenza Type A, Influenza Type B, SARS-CoV-2 and RSV

**Figure 3:**
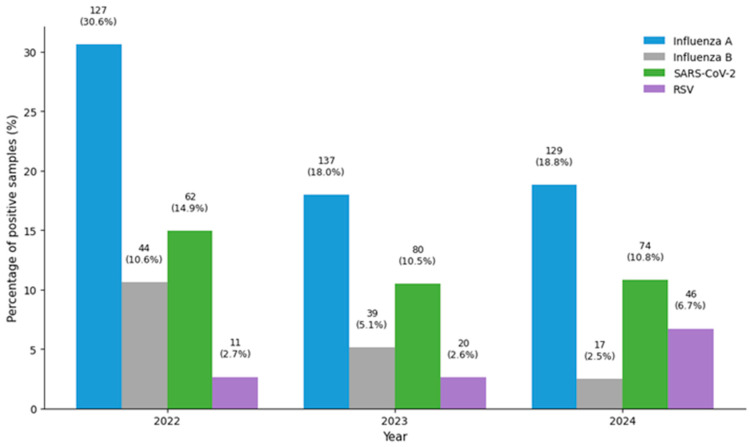
Number and percentage of positive samples for influenza type A, influenza type B, SARS-CoV-2 and RSV among tested samples (2022–2024).

A total of 1864 samples collected by the NCDC sentinel sites in Libya during 2022–2024 were tested for influenza type A, influenza type B, SARS-CoV-2, and RSV. Influenza virus was detected in 26.4% (493/1864) of samples [influenza type A was detected in 21.1% (393/1864) of samples and influenza B was detected in 5.4% of samples (100/1864)], SARS-CoV-2 was detected in 11.6% (216/1864) of samples, and RSV was detected in 4.1% (77/1864) of samples (Figure 3).

**Figure 4:**
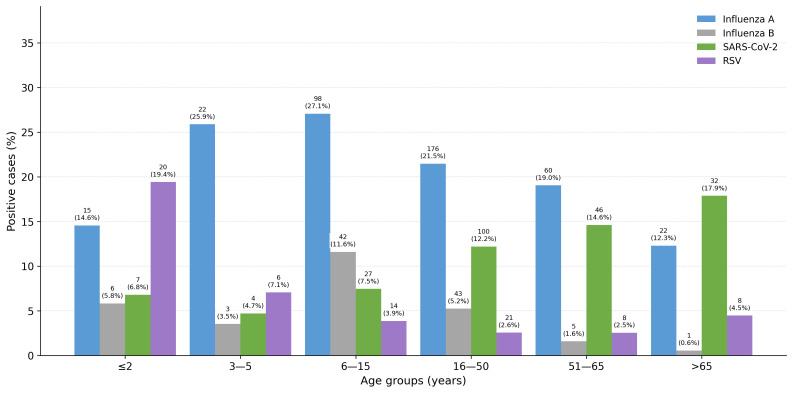
Number and percentage for each age group of positive samples for influenza type A, influenza type B, SARS-CoV-2 and RSV among tested samples (2022–2024).

The distribution of positive cases for influenza type A, influenza type B, SARS-CoV-2, and RSV across different patient age groups among tested samples (2022–2024) is shown in Figure 4.

The twenty-two previously described samples, which were initially positive for influenza A, as detected by GeneXpert, and stored at −80 °C, were selected for further analyses. Upon re-testing these samples at the NRCTTD and National Influenza Center of Tunisia, only fifteen of the samples were confirmed positive in both laboratories (Table 1).

### 3.2. Subtypes Identified on Confirmed Positive Samples

Of the 15 samples confirmed as positive at the National Influenza Center of Tunisia, the majority were identified as influenza A(H1N1) pdm09 (*n* = 11), whereas the remaining samples were classified as influenza A(H3N2) (*n* = 4). Eleven of these samples were subsequently selected for sequencing, including nine A(H1N1)pdm09 and two A(H3N2).

### 3.3. Sequencing Output

From the influenza A-positive samples, 11 were selected for whole-genome sequencing. Complete genome sequences covering all eight gene segments were successfully obtained for seven H1N1pdm09 isolates, while partial genome sequences were obtained for the remaining samples, including high-quality HA sequences from three additional H1N1pdm09 and two H3N2 isolates, as well as an NA sequence from one additional H1N1 isolate.

Genetic analysis showed that all A(H1N1)pdm09 strains belonged to clade 6B.1A.5a.2a (5a.2a), while the A(H3N2) strains were clustered within clade 3C.2a1b.2a.2a.3a.1 (2a.3a.1) based on the HA mutation profile. No mutations associated with antiviral resistance were detected based on the NA gene analysis.

### 3.4. Molecular Characterization

**Figure 5:**
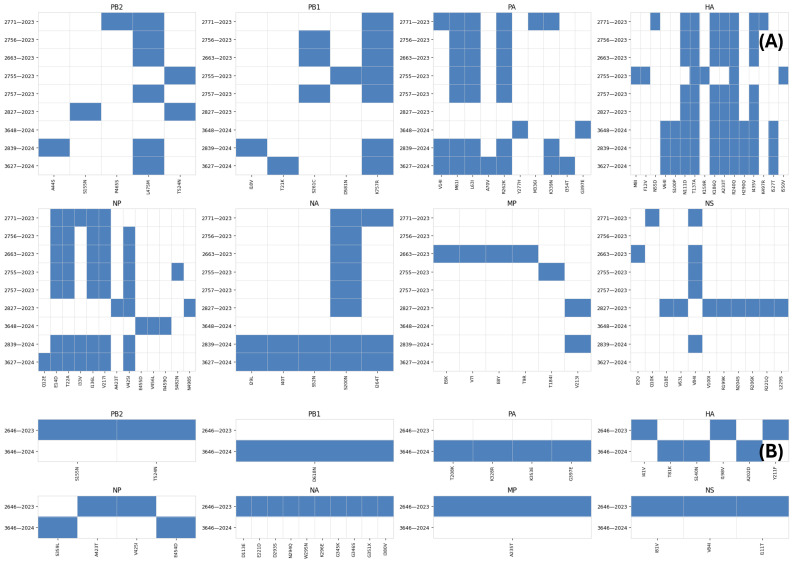
Heatmap of amino acid substitutions in eight segments of influenza A(H1N1)pdm09 (**A**) and H3N2 (**B**) viruses. Each row represents an individual virus sequence. Each column represents a specific amino acid position where mutations occurred. The presence of a mutation is indicated by a blue square, and an absence is indicated by a white square.

Molecular characterization based on amino acid substitution analysis performed using FluSurver (GISAID) revealed distinct mutation patterns in the HA and NA segments across different years and subtypes. For A(H1N1)pdm09 isolates, the HA segment showed limited amino acid substitutions in 2023, followed by the emergence of additional mutations in 2024, including T81K, S140N, and A202D. NA mutations were more prominent in the 2023 isolate, while fewer substitutions were observed in 2024, indicating variability among isolates rather than a consistent temporal trend. For A(H3N2) isolates, the HA segment exhibited lower genetic diversity compared to H1N1pdm09. The detailed amino acid substitutions identified are summarized in Figure 5.

While mutations were observed across isolates, they were less variable and more conserved within groups. The NA segment showed limited substitutions in 2023, followed by an increase in mutations in 2024 isolates. No known amino acid substitutions associated with reduced susceptibility to neuraminidase inhibitors were identified in the NA segment.

Whole-genome sequencing also demonstrated amino acid substitutions across additional viral proteins, including PB2, MP, NS, and PA-X, with some substitutions recurring among isolates. One isolate presented only partial NA sequence data, but overall, these findings reflect genome-wide genetic diversity among the sequenced IAVs.

This study represents the first submission of human IAV sequences from Libya to the GISAID database. The deposited sequences were assigned the following accession numbers: EPI_ISL_20311925 (A/Libya/3646/2024), EPI_ISL_20311731 (A/Libya/3627/2024), EPI_ISL_20311675 (A/Libya/2839/2024), EPI_ISL_20311593 (A/Libya/2771/2023), EPI_ISL_20311567 (A/Libya/2757/2023), EPI_ISL_20311527 (A/Libya/2756/2023), EPI_ISL_20311468 (A/Libya/2755/2023), and EPI_ISL_20311368 (A/Libya/2663/2023).

All genome sequences and associated metadata supporting the findings of this study can be accessed through the persistent digital object identifier https://doi.org/10.55876/gis8.260114oe.

### 3.5. Phylogenetic Analysis of the Hemagglutinin (HA) Gene Segment

Phylogenetic analysis of the HA gene was performed using two datasets comprising 118 H1N1 sequences (including seven A(H1N1)pdm09 Libyan isolates) sampled during 2021–2024 and 58 H3N2 sequences (including two Libyan isolates) sampled during 2022–2024, representing the global diversity of circulating viruses.

**Figure 6:**
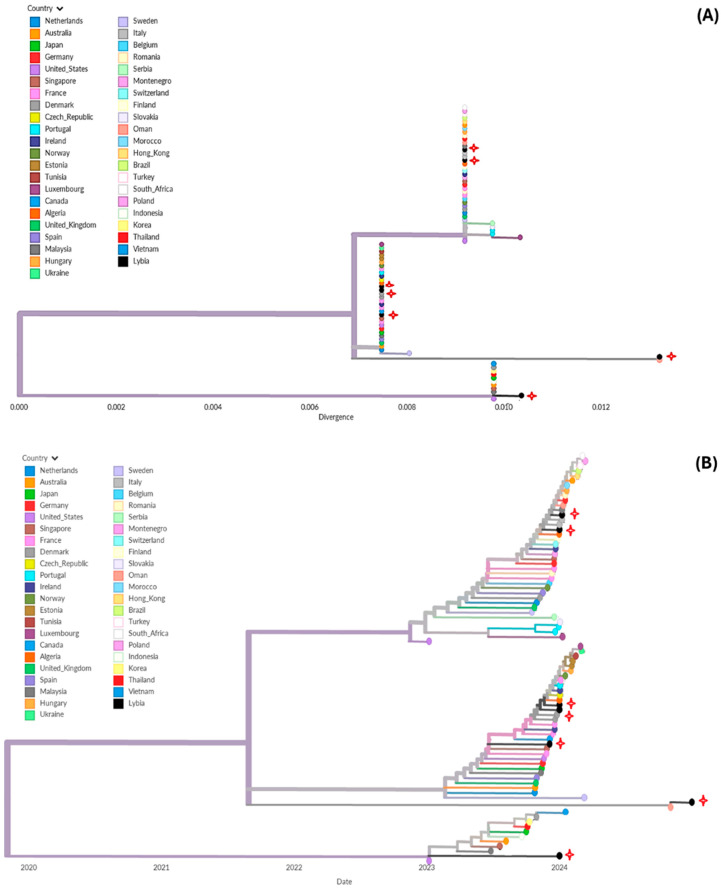
Global phylogenetic analysis of influenza A(H1N1)pdm09 viruses: (**A**) Genetic divergence tree showing the distribution of Libyan isolates within the global context. (**B**) Time-scaled phylogenetic tree illustrating the temporal relationships among sequences. Libyan strains are highlighted with red stars. Interactive versions of the trees are available at https://nextstrain.org/fetch/raw.githubusercontent.com/Sana-Ferjani/influenza_Lybien_tree/refs/heads/main/H1N1-1%20(1).json (accessed on 15 March 2026).

The A(H1N1)pdm09 phylogeny showed that Libyan isolates were clustered across multiple branches of the global tree. Genetic divergence analysis indicated that these isolates were distributed among three major clades. Clade I included sequences from geographically proximate countries such as Tunisia and Algeria, as well as several European countries. Clade II was predominantly composed of European sequences, with additional representation from South Africa, the United States, and parts of Asia. Clade III mainly comprised Asian sequences, including those from Singapore, Malaysia, Japan, Thailand, and Vietnam, along with sequences from Oman (Figure 6).

**Figure 7:**
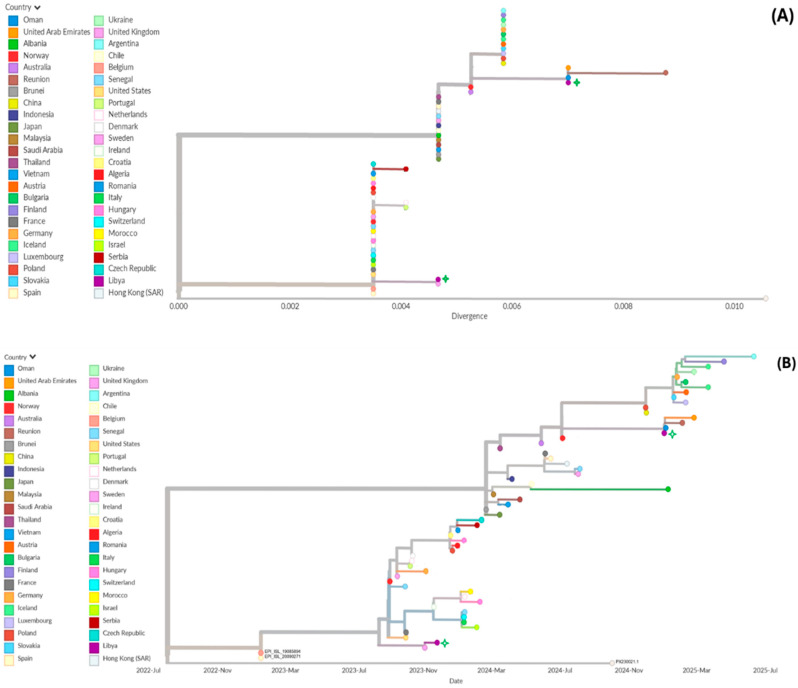
Global phylogenetic analysis of influenza A(H3N2) viruses: (**A**) Genetic divergence tree showing the placement of Libyan isolates within global diversity. (**B**) Time-scaled phylogenetic tree illustrating evolutionary relationships over time. Libyan strains are highlighted with green stars. Interactive versions of the trees are available at https://nextstrain.org/fetch/raw.githubusercontent.com/Sana-Ferjani/influenza_Lybien_tree/refs/heads/main/H3N2.json (accessed on 15 March 2026).

The Libyan A(H3N2) isolates were distributed across two distinct clades in the divergence-based phylogenetic analysis (Figure 7). One isolate was clustered with sequences predominantly from Europe, including a strain from Sweden, while the second was clustered with sequences reported from the Middle East, including Oman and the United Arab Emirates.

## 4. Discussion

### 4.1. Comparison of Influenza A Detection with Other Respiratory Viruses

Previous studies in Libya reported variable detection rates of influenza A across different epidemiological periods [20]. For example, only 2.8% of tested samples were positive for influenza A during the 2021–2022 season in Libya. This low detection rate was likely attributable to public health interventions implemented during the COVID-19 pandemic, including widespread mask use, reduced social gatherings, and increased public awareness of respiratory infection prevention. Collectively, these measures contributed to a substantial decline in the circulation of seasonal influenza viruses.

In contrast, Elahmer et al. [7] reported a markedly higher influenza A positivity rate (35%) during the 2009 H1N1 pandemic, reflecting the enhanced transmission dynamics typically observed during pandemic periods. At the regional level, influenza A has been reported to predominate, accounting for approximately 67% of influenza cases in the Eastern Mediterranean and North African regions [21]. Similar trends have been reported globally, with influenza A accounting for 91.8% of detected influenza cases in China between December 2022 and January 2023 [22].

In the present study, surveillance data from the NCDC demonstrated that influenza A was the most frequently detected respiratory virus during the 2022, 2023, and 2024 influenza seasons. An increasing trend in influenza A detection was observed across these seasons, indicating a clear resurgence of seasonal influenza activity following the COVID-19 pandemic. This pattern is consistent with the relaxation of non-pharmaceutical interventions, including reduced mask use and increased population mobility, which likely facilitated the re-emergence and sustained circulation of influenza viruses. The continued predominance of influenza A underscores its public health significance and highlights the need for sustained molecular surveillance, particularly in regions such as Libya, where influenza genomic data remain limited.

In addition to epidemiological factors, pre-analytical conditions likely influenced viral detection efficiency, as sample storage, handling, and transportation are critical determinants of RNA integrity [23]. In the present study, repeated freeze–thaw cycles and delays in sample processing may have contributed to RNA degradation. Although 22 samples were initially positive, only 15 yielded detectable viral RNA upon re-analysis. Additionally, cycle threshold (Ct) values were notably higher than those obtained during initial testing, indicating reduced viral RNA concentrations, likely due to RNA instability associated with suboptimal storage conditions. It was noted that there were discrepancies between GeneXpert results and confirmatory RT-PCR testing (NRCTTD and NIC Tunisia), including differences in Ct values and a lack of confirmation in some samples. These discrepancies can be explained by a combination of pre-analytical and methodological factors:

First, RNA degradation is likely a major contributor. The samples were stored at −80 °C and subsequently transported between laboratories, during which temperature fluctuations and multiple freeze–thaw cycles may have occurred. Such conditions can reduce RNA integrity [16], particularly in samples with lower viral loads, leading to increased Ct values or false-negative results upon re-testing—even in cases that initially showed relatively low Ct values.

Second, differences in diagnostic platforms and protocols may contribute to variability. The GeneXpert system is a highly sensitive, closed, cartridge-based platform with integrated extraction and amplification, whereas the confirmatory assays involved separate RNA extraction and RT-PCR steps, which may differ in sensitivity, efficiency, and target gene regions. These differences can result in variation in Ct values and detection outcomes.

Third, stochastic effects in samples with low or borderline viral RNA concentrations may also explain inconsistent detection across laboratories. These findings highlight the importance of minimizing freeze–thaw cycles and ensuring optimal sample preservation to enhance molecular detection sensitivity. In addition, significant logistical constraints impacted this research work. Due to the lack of local sequencing facilities, samples had to be transported to Tunis for sequencing. These challenges further limited the number of samples that could be successfully processed and sequenced.

These limitations reflect broader challenges in the influenza surveillance system in Libya, particularly regarding cold chain maintenance, logistics, and resource availability.

### 4.2. Subtyping of Influenza A

In this study, subtyping was performed on 18 influenza A-positive samples, representing the first molecular characterization of circulating human influenza A subtypes in Libya. The A(H1N1)pdm09 subtype predominated, accounting for approximately 72% of cases, while A(H3N2) comprised roughly 27% of cases.

Although previous studies in Libya have reported influenza A detection, most did not include subtype differentiation or were limited to non-human settings, highlighting the novelty of the present findings. The observed subtype distribution is consistent with reports from the Middle East and North Africa (MENA) regions [24], where A(H1N1)pdm09 has predominated in recent seasons, followed by A(H3N2). This pattern is further supported by the World Health Organization, which reported that A(H1N1)pdm09 was the most active influenza A subtype in North Africa between September 2023 and January 2024 [12]. In contrast, surveillance data from Tunisia during the 2022 influenza season, derived from patients admitted to the medical intensive care unit (MICU), indicated that influenza A(H3N2) was the dominant circulating subtype [25]. This finding highlights regional variability in influenza subtype circulation and suggests that H3N2 may be more strongly associated with severe disease rather than higher community prevalence, potentially influenced by differences in population immunity, viral evolution, and local epidemiological dynamics.

### 4.3. Sequencing of H1N1 and H3N2

According to the GISAID platform, multiple genetic clades have circulated globally in recent influenza seasons, including several subclades of 6B.1A for A(H1N1)pdm09 and predominantly 3C.2 and its subclades for A(H3N2). Notably, no human influenza A sequences from Libya were available in GISAID during these periods, highlighting the scarcity of publicly available genomic data and underscoring the importance of the present study. Whole-genome sequencing in this work demonstrated that all Libyan A(H1N1)pdm09 strains belonged to clade 6B.1A.5a.2a, whereas the A(H3N2) strains clustered within clade 3C.2a1b.2a.2a.3a.1 were based on hemagglutinin (HA) mutation profiles.

Molecular analysis of the neuraminidase (NA) gene identified multiple amino acid substitutions; however, none corresponded to well-established neuraminidase inhibitor resistance-associated mutations. Recurrent substitutions, including S200N, I264T, and S52N, likely represent natural polymorphisms, and mutations at positions 294–296 differed from the classical resistance-associated N294S substitution [26]. These findings suggest that the analyzed strains remain susceptible to neuraminidase inhibitors and emphasize the need for continued surveillance.

In addition, amino acid substitutions were detected in several internal proteins, including PB2, MP, and NS. These changes were scattered and recurrent among some isolates, consistent with natural genetic diversity and ongoing viral evolution, and no mutations previously associated with increased virulence or antiviral resistance were identified [11,26].

### 4.4. Phylogenetic Analysis Discussion

The A(H1N1)pdm09 phylogeny showed that Libyan isolates were distributed among multiple clusters within the contemporary global diversity and were closely related to sequences reported from different geographic regions, including Europe. In contrast, the A(H3N2) phylogeny showed a more restricted distribution, with Libyan isolates clustering within a limited number of lineages and showing genetic similarity to sequences reported from Europe and the Middle East.

These observations should be considered exploratory. Given the limited number of Libyan sequences and the absence of systematic sampling, the findings do not allow robust phylogeographic inference or clear determination of introduction routes. The observed clustering with sequences from neighboring countries and other regions may reflect broader regional and global circulation patterns rather than specific transmission dynamics.

Overall, our study highlights the need for expanded and systematic molecular surveillance to better characterize the genetic diversity and circulation patterns of influenza viruses in Libya.

This study also highlights the utility of genomic surveillance platforms, such as Nextstrain, for exploring transmission pathways and contextualizing local strains within global diversity. However, given the limited number of Libyan sequences analyzed, these observations should be interpreted with caution. The observed genetic similarity between Libyan and European strains may indicate that Northern Hemisphere vaccine recommendations are applicable to Libya, although further genomic data are needed to confirm this and better understand influenza virus dynamics in the region [12,17,22,23].

This study provides the first structured sentinel surveillance and molecular characterization of influenza A viruses in Libya, generating essential baseline epidemiological and genomic data from a previously underrepresented setting. Overall, these findings fill a critical geographic gap in global datasets, strengthen national surveillance capacity, and highlight the importance of sustained genomic monitoring to track viral evolution and enhance pandemic preparedness.

This study has several limitations, including the relatively small number of samples subjected to whole-genome sequencing and potential geographic and temporal sampling constraints. Future studies should aim to emphasize the need for expanded genomic surveillance, improve sample handling infrastructure, and integrate epidemiological and genomic data to better understand influenza dynamics in Libya.

## 5. Conclusions

During the recent influenza seasons, influenza A was the most prevalent among the detected respiratory viruses. Among influenza A-positive samples, the H1N1pdm09 subtype predominated, followed by H3N2, and no other influenza A subtypes were detected. Genetic sequencing of the influenza virus samples revealed no unusual or previously unreported mutations, and the analyzed viral isolates did not exhibit mutations associated with antiviral resistance. Comparative sequence analysis further demonstrated a high degree of genetic similarity between the strains identified in this study and the vaccine strain used in Libya during the study period.

Despite the limited number of analyzed samples, this study represents the first molecular and genetic characterization of human IAVs in Libya and provides baseline data that can serve as a reference for future surveillance and research. The findings underscore the importance of continuous molecular monitoring of influenza viruses in humans and animals to detect emerging variants, track viral evolution, and identify potential antiviral resistance at an early stage.

## Figures and Tables

**Table 1:** Details and Ct values of 22 selected influenza A samples tested and reconfirmed by RT-PCR.

NCDC CODE	Age	Gender	Collection Date	FLU A1 Ct	FLU A Ct NRCTTD	Flu A Ct CN	Flu Subtype
2651	48	F	21/11/2023	28.9	NEG	NEG	-
2646	7	M	21/11/2023	22.1	28	29	H3N2
2663	33	F	29/11/2023	18.3	29	30	A(H1N1)pdm09
2681	58	M	05/12/2023	20.1	28	35	A(H1N1)pdm09
2757	21	F	26/12/2023	20	33	32	A(H1N1)pdm09
2755	50	F	26/12/2023	24.1	31	32	A(H1N1)pdm09
2756	18	M	26/12/2023	15.7	30	27	A(H1N1)pdm09
2771	15	F	26/12/2023	23.3	32	33	A(H1N1)pdm09
2777	45	F	26/12/2023	20.2	NEG	NEG	-
2769	9	F	26/12/2023	33	NEG	NEG	-
2765	47	M	26/12/2023	24.8	NEG	NEG	-
2786	60	M	27/12/2023	22.1	NEG	NEG	-
2849	45	F	02/01/2024	32	NEG	NEG	-
2839	90	F	02/01/2024	14.8	29	28	A(H1N1)pdm09
2827	23	F	02/01/2024	21.8	NEG	37	A(H1N1)pdm09
3620	75	F	17/12/2024	24.8	35	35	H3N2
3623	45	M	22/12/2024	24.3	34	35	A(H1N1)pdm09
3627	29	F	25/12/2024	19.4	32	31	A(H1N1)pdm09
3646	60	F	29/12/2024	16.6	30	31	H3N2
3634	58	F	29/12/2024	27.5	35	35	H3N2
3648	40	M	31/12/2024	17.1	33	34	A(H1N1)pdm09
3654	48	M	31/12/2024	18.9	36	NEG	-

CN: Charles Nicolle Laboratory.

## Data Availability

The original contributions presented in this study are included in the article. Further inquiries can be directed to the corresponding author.

## Abbreviations

The following abbreviations are used in this manuscript:

NCDC	National Center for Disease Control
IAV	Influenza A virus
IAVs	Influenza A viruses
RSV	Respiratory syncytial virus
SARS-CoV-2	Severe acute respiratory syndrome-coronavirus-2
GISAID	Global Initiative on Sharing All Influenza Data
HA	Hemagglutinin
NA	Neuraminidase
